# Mechanisms of Adiponectin Action: Implication of Adiponectin Receptor Agonism in Diabetic Kidney Disease

**DOI:** 10.3390/ijms20071782

**Published:** 2019-04-10

**Authors:** Yaeni Kim, Cheol Whee Park

**Affiliations:** 1 Division of Nephrology, Department of Internal Medicine, College of Medicine, The Catholic University of Korea, 222, Banpo-daero, Seocho-gu, Seoul 06591, Korea; yaenikim82@gmail.com; 2 Institute for Aging and Metabolic Diseases, College of Medicine, The Catholic University of Korea, Seoul 06591, Korea

**Keywords:** adiponectin, metabolism, AdipoRon, lipotoxicity

## Abstract

Adiponectin, an adipokine secreted by adipocytes, exerts favorable effects in the milieu of diabetes and metabolic syndrome through its anti-inflammatory, antifibrotic, and antioxidant effects. It mediates fatty acid metabolism by inducing AMP-activated protein kinase (AMPK) phosphorylation and increasing peroxisome proliferative-activated receptor (PPAR)-α expression through adiponectin receptor (AdipoR)1 and AdipoR2, respectively, which in turn activate PPAR gamma coactivator 1 alpha (PGC-1α), increase the phosphorylation of acyl CoA oxidase, and upregulate the uncoupling proteins involved in energy consumption. Moreover, adiponectin potently stimulates ceramidase activity associated with its two receptors and enhances ceramide catabolism and the formation of its anti-apoptotic metabolite, sphingosine 1 phosphate (S1P), independently of AMPK. Low circulating adiponectin levels in obese patients with a risk of insulin resistance, type 2 diabetes, and cardiovascular diseases, and increased adiponectin expression in the state of albuminuria suggest a protective and compensatory role for adiponectin in mitigating further renal injury during the development of overt diabetic kidney disease (DKD). We propose AdipoRon, an orally active synthetic adiponectin receptor agonist as a promising drug for restoration of DKD without inducing systemic adverse effects. Its renoprotective role against lipotoxicity and oxidative stress by enhancing the AMPK/PPARα pathway and ceramidase activity through AdipoRs is revealed here.

## 1. Introduction

With the advent of modern conveniences promoting increased dietary ingestion and a sedentary lifestyle, it is inevitable that a higher proportion of the population is exposed to a state of energy excess that contributes to the exponential growth of diabetes and obesity-related diseases [1]. White adipose tissue stores energy in the form of triglycerides during nutritional affluence, but as its storage capacity becomes saturated, excess fat is redirected to non-adipose tissues, entering alternative non-oxidative pathways and promoting the organ-specific production of toxic lipid metabolites [2].

Derangements in lipid metabolism play a crucial role in the development and progression of diabetic kidney disease (DKD). The accumulation of free fatty acids, which are otherwise used as an energy source, in glomerular and tubular epithelial cells of diabetic kidneys indicates a state of energy surplus. This altered energy balance leads to lipotoxicity in the kidney, which is characterized by the deposition of fatty acid metabolites such as triglycerides, diacylglycerols, and ceramides, leading to intrarenal toxicity and cell death [3].

The role of adipose tissue or adipocytes as an endocrine organ secreting various adipokines, in particular adiponectin, has come to the forefront in the fight against diabetes and metabolic syndrome because it has been demonstrated to exert pro-metabolic effects through the modulation of glucose and lipid homeostasis both directly, in an organ-specific manner, and indirectly, by systemic amelioration of insulin sensitivity [4]. Indeed, the increased circulating adiponectin levels in patients with end-stage renal disease [5] and the increased expression of adiponectin receptors that positively correlates with serum and urinary adiponectin levels in rats with chronic renal failure [6] indicate that there might be an intriguing link between adiponectin and the kidney in the setting of renal injury. In this review, we deal with the molecular signaling pathways involved in adiponectin and its receptor binding, placing an emphasis on the recent progress in research on the role of the adiponectin receptor agonist, AdipoRon, in DKD.

## 2. Adiponectin in Renal Physiology: Its Association with Albuminuria and Glomerular Filtration Rate

Adiponectin circulates in a combination of three forms: (1) low-molecular-weight trimers that oligomerize to form (2) middle-molecular-weight hexamers that in turn agglomerate to form (3) high-order structures of oligomers of up to 800 kDa that potentiate the strongest insulin-sensitizing activity in hepatocytes [7]. Circulating adiponectin is primarily eliminated by the liver, and secondarily by the kidneys [8]. Since adiponectin monomers (28 kDa) and dimers are small enough to cross the glomerular filtration barrier, they can be detected in the urine of healthy individuals [9], whereas high-molecular-weight adiponectin has been reported to be excreted to a considerable extent in the urine of albuminuric and proteinuric patients, possibly as a result of leakage through a dysfunctional glomerular filtration barrier [10]. 

The association between plasma adiponectin concentration and urinary adiponectin excretion rate in proteinuric patients with or without diabetes is unclear. In those with low-grade microalbuminuria, a study involving subjects mainly of obese and metabolic syndrome backgrounds with a preserved glomerular filtration rate (GFR) found that urinary adiponectin and albumin excretion rates were negatively correlated with the plasma adiponectin level [11,12,13]. In contrast, a study population consisting of diabetics with reduced GFR and macroalbuminuria demonstrated a positive relationship between circulating adiponectin levels and urinary adiponectin and albumin excretion rates [5,14]. In a subpopulation analysis, an inverse relationship between the serum adiponectin level and urinary protein excretion rate in patients with type 2 diabetes with preserved GFR was attributed to the decrease in serum adiponectin associated with increased insulin resistance [15], whereas an increase in serum adiponectin levels has been established in patients with type 1 diabetes [16]. This increase in urinary or serum adiponectin concentration is not in its receptor-bound form and thus it is not metabolically active. Markedly increased adiponectin has been consistently reported in patients with both chronic kidney disease (CKD) and end-stage renal disease (ESRD), and this upregulation of circulating adiponectin has been deemed a compensatory mechanism to relieve further renal injury and subsequent unbound form adiponectin in excess may have been filtered through loosened and defective glomerular filtration barrier and excreted in the urine [17,18].

Several contradictory observations have been reported in the literature to date. It is hard to interpret the adiponectin-albuminuria association in the setting of DKD since impaired GFR frequently coexists that tends to increase circulating adiponectin level, and its impact cannot be investigated separately from the presence of albuminuria. Moreover, the effect of antidiabetic regimen that patients might have been following could also influence circulating adiponectin levels, further hindering the identification of a causal relationship. Collectively, an increase in serum adiponectin together with decreased urinary adiponectin and albumin excretion rates and decreased GFR point towards a renoprotective role for adiponectin in decreasing its urinary loss and preserving renal function. Nevertheless, it is important to balance these strong clinical correlations with consideration of whether alterations in adiponectin are always a cause or a consequence of disease states, and thus, it is likely that the timing of targeted adiponectin therapy will be vital to its success in this metabolic milieu.

## 3. Expression of Adiponectin and Its Receptors and Their Implication for Renoprotection

In the kidney, adiponectin is found on the endothelium of the glomerular and peritubular capillaries, on the smooth muscle cells of intrarenal arteries/arterioles, and proximal and distal tubular epithelial cells [19,20]. It has been reported that a fair amount of AdipoR1 is expressed in the cells constituting the glomerulus: endothelial cells, podocytes, mesangial cells, and Bowman’s capsule epithelial cells, as well as in proximal tubular cells, whereas AdipoR2 is expressed to a lesser degree on glomeruli and proximal tubular cells [20,21].

The renoprotective properties of adiponectin through binding to its receptors have been implicated in several rodent models. Sharma et al. demonstrated that adiponectin knockout mice exhibited increased albuminuria and segmentally fused podocyte foot processes that were restored by adiponectin administration. In addition, albumin permeability across a differentiated podocyte cell monolayer was reduced by the addition of adiponectin in vitro. 5-aminoimidazole-4-carboxamide-1-β-d-ribonucleoside (AICAR), a specific activator of AMPK, reduced the permeability of podocytes to albumin, whereas adenine 9-β-d-arabinofuranoside, a specific inhibitor of AMPK, increased the permeability of podocytes to albumin, suggesting a protective role of adiponectin against the development of albuminuria, at least in part, through the direct action of adiponectin-induced activation of the AMPK pathway in podocytes. This was independent of adiponectin’s systemic effect, since AMPK activation in podocytes was induced specifically by restoring the localization of zona occludens-1 along the plasma membrane of podocytes, which contributed to the podocytes’ structural and functional integrity associated with tight junction adherence and the narrowing of the slit diaphragm [21].

Rutkowski et al. generated a mouse model that allowed the induction of caspase-8-mediated apoptosis specifically in podocytes (POD-ATTAC mice). POD-ATTAC mice lacking adiponectin developed significant albuminuria and ablated podocytes; however, adiponectin-overexpressing POD-ATTAC mice recovered renal function and ameliorated podocyte injury and interstitial fibrosis, suggesting that adiponectin helped to reverse podocyte injury and restore renal function [22].

Fang et al. confirmed the hypothesis that adiponectin may attenuate the deleterious effects of angiotensin II in renal tubular cells by showing that angiotensin II-induced nicotinamide adenine dinucleotide phosphate (NADPH) oxidase activation and oxidative stress were attenuated by AdipoR1 activation. Activation of AMPK with AICAR mimicked the effect of adiponectin on angiotensin II-induced activation of NADPH oxidase. Angiotensin II-induced activation of NADPH oxidase was abrogated by coincubation with the AMPK inhibitor compound C, indicating that the renoprotective effect of adiponectin binding to AdipoR1 was achieved through the subsequent activation of AMPK [23].

Yu et al. created a chronic renal failure rat model by adenine administration that exhibited increased serum and urinary adiponectin levels that positively correlated with the intrarenal expression of both AdipoR1 and AdipoR2. Significant upregulation of the expression of adiponectin and its receptors might be reflective of an adaptive renal response to compensate for ongoing renal injury [6]. These data suggest that adiponectin’s effect on its specific target organ might be achieved by binding to its receptors in the relevant tissues, independently of its systemic effect. The above-mentioned studies investigating the renoprotective effects of adiponectin concerning podocyte recovery were carried out exclusively in rodent experimental settings. Therefore, the relevance of these studies to the human situation in different pathological backgrounds should be interpreted with caution.

Our study investigating the expression of adiponectin receptors in human diabetic kidneys demonstrated significantly decreased expression of AdipoR1 and AdipoR2, even at an early stage of CKD, that was maintained throughout the progression of CKD stages compared to that of non-diabetic kidneys, and this down-regulation of adiponectin receptors might be in part due to the increased insulin resistance in diabetes [24,25]. This is in keeping with the evidence that obesity decreases not only plasma adiponectin levels but also AdipoR1/R2 expression, thereby reducing adiponectin sensitivity and leading to insulin resistance, which in turn aggravates hyperinsulinemia [26]. This suggests that both upregulation of AdipoR1 and AdipoR2 expression and agonism of AdipoRs could be potential targets for novel treatments for insulin resistance and type 2 diabetes.

## 4. Signaling Pathways Associated with Adiponectin and Its Receptor Binding

Adiponectin exerts its effects via binding to three receptors: AdipoR1, AdipoR2, and T-cadherin. AdipoR1 and AdipoR2 have seven transmembrane domains and are significantly homologous, sharing 67 % amino acid identity [27], whereas T-cadherin is considered to be an adiponectin-binding protein but its functional significance has not been completely determined. It is thought that the latter receptor has no effect on adiponectin’s cellular signaling or function, since it does not have an intracellular domain [28]. The binding of adiponectin to its receptors can regulate glucose and lipid homeostasis by promoting a strong insulin-sensitizing effect, fatty acid oxidation, mitochondrial biogenesis, and mediating anti-oxidative and anti-inflammatory effects. AMPK and PPARα are primary targets activated by AdipoR1 and AdipoR2, respectively [29,30].

AMPK is a metabolic master switch that regulates downstream signals based on shifts in the surrounding energy reservoir [31]. AMPK activation can be triggered as a result of the conformational change incurred by an adenosine monophosphate (AMP) binding to its γ subunit and phosphorylation of the α subunit by upstream kinases, including a compound consisting of three proteins: STE-related adaptor (STRAD), mouse protein 25, and the tumor-suppressor liver kinase B1 (LKB1) [32]. LKB1 activation and calcium influx-induced activation of Ca^2+^/calmodulin-dependent protein kinase kinase β (CaMKKβ) are triggered upon AdipoR1 activation that primarily potentiates AMPK stimulation [33]. Upon activation, AMPK signals through its downstream substrates to achieve energy homeostasis by stimulating processes that generate ATP through actions such as glucose transport, mitochondrial biogenesis, and fatty acid oxidation, while inhibiting those that use ATP through the opposing actions of fatty acid, protein, and glycogen synthesis [31].

AMPK modulates glucose transport in a similar way to insulin. AMPK promotes glucose uptake in peripheral tissues by promoting GLUT4 translocation to the cell membrane and upregulating the expression of hexokinase II [34]. Hyperglycemia-induced oxidative stress upregulates vascular endothelial growth factor expression in podocytes that increases vascular permeability and activates classical pathways associated with the production of advanced glycosylation end products and the activation of protein kinase C and aldose reductase that contribute to the development of DKD through its characteristic pathological changes in mesangial cell proliferation and hypertrophy, exacerbated matrix production, and basement membrane thickening [35,36]. Therefore, targeting AMPK could ameliorate these adverse effects by enhancing insulin sensitivity at the systemic level and regulating glucotoxicity-induced oxidative stress in the target organ as well.

AMPK is also known to mediate the intracellular signaling pathway of class O forkhead box (FoxO) proteins. FoxO proteins are transcription factors that regulate the expression of antioxidant enzymes; promote mitochondrial biogenesis, cell survival, and longevity in several tissues; and participate in tumor suppression [37]. The transcriptional activity of the subfamily member FoxO3a is modulated by AMPK in response to metabolic stress to shield quiescent cells from reactive oxygen species (ROS) by antagonizing apoptosis, which reduces oxidative stress by directly increasing the quantity of antioxidant enzymes such as thioredoxin, peroxiredoxin, manganese superoxide dismutase (SOD), and catalase [38]. Therefore, when activated upon ROS exposure, the AMPK-FoxO3a signaling pathway upregulates the expression of silent information regulator T1 (SIRT1), a well-known FoxO3a coactivator, that is thought to mediate apoptotic and autophagy crosstalk [39]. Our previous study investigating the effect of resveratrol suggested that resveratrol can ameliorate renal cell apoptosis and oxidative stress via activation of the AMPK-SIRT1-PGC1α axis and its consequent effects on the phosphatidylinositol-3 kinase (PI3K)-Akt (protein kinase B)-FoxO3a pathway, which induces mitochondrial biogenesis and enhances its capacity to alleviate oxidative stress in DKD [40]. The renoprotective role of human recombinant extracellular superoxide dismutase (EC-SOD) on DKD—amelioration of hyperglycemia-induced oxidative stress, inflammation, and apoptosis through the activation AMPK-PGC1α-nuclear factor erythroid 2-related factor 2 (Nrf2) and AMPK-FoxOs pathways—was also demonstrated in our recent study [41].

The AMPK dependent phosphorylation of downstream target substrates involved in regulating protein translation, cell growth, and autophagy includes tuberous sclerosis complex protein-2 (TSC2) and mammalian target of rapamycin (mTOR) complex 1 that repress protein synthesis and deliver their renoprotective effect by inhibiting the accumulation of extracellular matrix in DKD [42].

AMPK modulates changes in lipid metabolism via the regulation of fatty acid oxidation and cholesterol synthesis in the target organ. AMPK phosphorylation decreases the activity of the lipogenesis-associated enzymes sterol regulatory element-binding protein-1 (SREBP-1), acetyl CoA carboxylase-1 (ACC-1), and hydroxymethylglutaryl CoA reductase (HMGCR), which in turn limit malonyl CoA production, attenuating the inhibition of carnitine palmitoyl transferase-1 (CPT-1) activity that potentiates the transport of fatty acids into the mitochondria for beta oxidation and interrupts fatty acid synthesis [43].

Moreover, AdipoR2-induced activation of PPARα promotes fatty acid catabolism by upregulating genes involved in fatty acid transport, binding and activation, and peroxisomal and mitochondrial fatty acid β-oxidation. PPARα-mediated gene transcription upregulates the PGC1α and ERR1α axis that enhances mitochondrial oxidative capacity to reduce oxidative stress and further contributes to decreased lipid accumulation in the target organ [44]. As a result, the increased endothelial nitric oxide synthase (eNOS) level is expected to neutralize ROS, reduce adhesion molecule synthesis, and suppress cell proliferation, which collectively help to confer protective effects on endothelial cells against albuminuria development in the kidney [24]. We recently unraveled a causal relationship between lipotoxicity and lymphangiogenesis using the PPARα agonist fenofibrate that improved intrarenal lipotoxicity and secondary lymphatic proliferation through PPARα-AMPK-pACC signaling in DKD [45].

Adiponectin modifies the effects of toxic ceramide accumulation by binding to AdipoR1/2, which increases ceramidase activity, catalyzing the conversion of ceramide to sphingosine and subsequently sphingosine-1 phosphate (S1P), independently of AMPK [46]. Ceramides consist of sphinosines and fatty acids that make up sphingomyelin, one of the major lipids in the bilayer of the cell membrane. In addition to its original role as a structural element, sphingomyelin acts as a cellular signaling molecule and regulates the differentiation, proliferation, programmed cell death, and apoptosis of cells [47]. Among its different subtypes, ceramide (C)16, C18, and C24 are associated with adverse clinical outcomes with their fatty acid components accumulating in the target organ promoting cell apoptosis and insulin resistance [48]. Surplus fatty acids in target organ cells stimulate the transcription of enzymes involved in ceramide biosynthesis that disrupt the association between inhibitor 2 of protein phosphatase 2A (I2PP2A) and protein phosphatase 2A (PP2A) [49]. PP2A impairs NO bioavailability by promoting the dephosphorylation and inactivation of Akt and B-cell lymphoma 2 (Bcl-2), leading to endothelial dysfunction and apoptosis, respectively [50,51]. Consequently, increased ceramidase activity results in a net increase in the S1P to ceramide ratio that promotes three key metabolic effects: it provides sphingosine and free fatty acids as ligands for PPARα, promotes the formation of the pro-survival factor S1P, and decreases ceramide accumulation that further increases insulin sensitivity and NO production. This is in line with our recent finding that AdipoR activation may ameliorate intrarenal triglyceride accumulation and endothelial dysfunction through increased ceramidase activity and the subsequent normalized ceramide to S1P ratio in DKD [25].

The studies discussed above are only a handful of many that describe the activation of AMPK, PPARα, and enhanced ceramidase activity through AdipoR binding. Highlighting the concept of lipotoxicity as a main contributor to the development of DKD, it is noteworthy that AdipoR1 and AdipoR2 serve as receptors for globular and full-length adiponectin that are primarily involved in cellular signaling pathways promoting mitochondrial oxidative capacity and fatty acid oxidation. This raises the potential for AdipoR activation via adiponectin binding as a therapeutic target for optimizing lipid metabolism in DKD.

## 5. Adiponectin and Adiponectin Receptors as Therapeutic Targets for DKD

### 5.1. Strategy for Upregulation of Adiponectin and Its Receptors

The general consensus based on the available literature indicates that adiponectin is renoprotective and, therefore, it seems a logical approach that antidiabetic therapy should be aimed at enhancing adiponectin effects by increasing the plasma level of adiponectin itself or by activating adiponectin receptors to increase adiponectin sensitivity, which subsequently would render pleiotropic metabolic effects via their downstream signaling pathways.

Pravastatin therapy significantly improves insulin sensitivity and increases plasma adiponectin levels in patients with hypercholesterolemia and in those with coronary heart disease with impaired glucose tolerance. The mechanism involving HMG-CoA reductase inhibition may account for the reported increased plasma adiponectin level via which this particular statin delivers its therapeutic effect [52]. Blocking the renin-angiotensin-aldosterone system (RAAS) by either physiologic or pharmacologic modulation of RAAS activity, e.g., by using enalapril or valsartan, increased adiponectin production and upregulated circulating adiponectin. Valsartan blocks the constitutive angiotensin II type 1 receptor activity involving the nuclear factor κB (NF-κB) pathway that limits PPARγ activity in mature adipocytes, thus attenuating the proinflammatory response and enhancing the insulin-sensitizing activities of mature adipocytes, which may underlie the beneficial metabolic impact of angiotensin II receptor blockers (ARBs) [53]. Increasing the level of adiponectin using RAAS blockers might improve the anti-inflammatory response in DKD by activating the AMPK and cyclooxygenase-2 pathways, and decreasing tumor necrosis factor-α (TNF-α) activity. Adiponectin has also been shown to inhibit angiotensin II–induced activation of NADPH oxidase via the AdipoR1-mediated activation of both AMPK and cAMP-Epac pathways [54]. Tesaglitazar is a PPARα/γ dual agonist that exerts its favorable effect by increasing plasma adiponectin levels. It not only improved insulin resistance and lipid metabolism at the systemic level, but also prevented albuminuria and renal glomerular fibrosis in a diabetic mouse model, proving its potential as a promising antidiabetic agent for DKD [55]. Fenofibrate has been reported to increase adiponectin expression in adipose tissue and serum adiponectin levels through PPARα activation and the elevation of high-density lipoprotein levels [56]. Salsalate treatment demonstrated metabolic improvements in terms of increased insulin sensitivity and lipid profiles in obese Hispanics by significantly increasing serum adiponectin levels, which occurred without alterations in adiposity [57]. Thiazolidinediones (TZD), e.g., pioglitazone and rosiglitazone, have been shown to directly upregulate adiponectin gene transcription via activation of PPARγ in adipose tissue, thereby promoting adipocyte differentiation and/or increasing the number of small adipocytes that are more sensitive to insulin [58,59]. However, whether the TZD-induced increase in the plasma adiponectin level is causally involved in the TZD-mediated insulin-sensitizing effects has not been addressed experimentally. Rosiglitazone, a PPARγ agonist, not only improved metabolic parameters encompassing plasma adiponectin, fasting glucose, glucose metabolic clearance rate, and TNF-α, but also alleviated albuminuria, suggesting its potency as a renoprotective agent for T2D patients [60]. It has also become clear that a fasted/starved state, caloric restriction, and/or weight loss lead to increases in circulating levels of adiponectin, and that SIRT1 increases adiponectin or inhibits inflammatory cytokines by deacetylating PPARγ [61].

However, there are significant side-effects associated with chronic adiponectin upregulation. Notably, mice genetically engineered to overexpress adiponectin and those treated with molecules that stimulate adiponectin secretion, such as thiazolidinediones and fibroblast growth factor 21, showed reduced bone density [62]. Heart damage—left ventricular hypertrophy in particular—was one of the other adverse effects that has been observed in rodents upon chronic increase in adiponectin production [63,64]. Adiponectin may also promote adipogenesis and angiogenesis associated with weight gain and the growth of tumors, respectively [65]. Lastly, infertility can be triggered by chronically elevated adiponectin concentrations [66]. The mechanism by which chronic adiponectin exposure mediates detrimental effects on various tissues is unclear. However, experimental evidences indicate that its innate properties to self-associate into higher-order structures with high turn-over rate and sexual dimorphism may attribute to the yet unraveled defaults associated with chronic adiponectin exposure [8]. Moreover, in case of its association with reduced bone mass, it is implicated that circulating adiponectin especially in its full-length form, rather than globular form, indirectly inhibits bone mass by increasing insulin sensitivity and inhibiting the action of insulin in tissues [67,68]. These potential pitfalls will need to be addressed with deliberation when establishing a strategy exploiting the upregulation of adiponectin and its receptors.

### 5.2. Development of an AdipoR Agonist, AdipoRon

Okada-Iwabu et al. identified several molecules that activate adiponectin receptors and focused their in-depth analysis on devising an orally active synthetic compound called “AdipoRon.” AdipoRon binds, at a low micromolar concentration, to both AdipoR1 and AdipoR2. It is capable of producing the pro-metabolic effects of adiponectin by binding to both AdipoR1/2 and subsequently activating AMPK, PPARα, and the transcriptional coactivator PGC1α, which boost mitochondrial proliferation and energy metabolism [69]. When diabetic mice fed a high-fat diet were treated with AdipoRon, the metabolic improvements, including enhanced glucose and lipid metabolism and insulin sensitivity, were conferred in the liver and skeletal muscle, which ultimately increased their exercise endurance capacity and extended their life span [69]. Overexpression of the oxidative stress-relieving genes catalase and SOD might have led to the increase in lifespan [70]. Despite the aforementioned concerns, AdipoRon did not promote weight gain in mice, and the reported observation of a prolonged life span in diabetic mice helps to obviate some of these concerns, even though many of these effects require a more chronic exposure to be manifested. Although the studies described focused on the endocrine effects of AdipoRon, it is again important to consider the potential local effects of AdipoRon in the target organ that is the diabetic kidney.

### 5.3. Role of AdipoRon in DKD

In human diabetic kidneys, the expression of AdipoR1, AdipoR2, and CaMKKβ, and the number of phosphorylated LKB1- and AMPK-positive cells significantly decreased when compared to those of controls and the extent of glomerulosclerosis and tubulointerstitial fibrosis correlated with renal functional deterioration. In a diabetic mouse model, AdipoRon directly activated intrarenal AdipoR1 and AdipoR2, which increased CaMKKβ, phosphorylated Ser^431^LKB1, phosphorylated Thr^172^AMPK, and PPARα expression independently of the systemic effects of adiponectin. AdipoRon also decreased intrarenal ceramide species and restored the activity of acid ceramidase, the concentration of S1P, and the ratio of ceramide to S1P. In vitro studies confirmed that AdipoRon increased the intracellular Ca^2+^ influx that activated the CaMKKβ/phosphorylated Ser^431^LKB1/phosphorylated Thr^172^AMPK/PPARα pathway and downstream signaling, thus decreasing oxidative stress and apoptosis and improving endothelial dysfunction in human glomerular endothelial cells and murine podocytes treated with high-glucose- and palmitate-media. Adiponectin receptor agonism recovered podocytes’ structural integrity as demonstrated by increased slit-diaphragm diameter and decreased foot process width, and glomerular basement membrane (GBM) thickness in electron microscope (EM) findings. We also confirmed favorable impact of AdipoRon on renal phenotype; it ameliorated features of DKD through decreased intrarenal fibrosis, inflammation and apoptosis as shown by decreased mesangial fraction area and expression of collagen IV, TGF-β, and F4/80 on PAS and immunohistochemical staining. Collectively, AdipoRon treatment exerted renoprotective effects by improving diabetes-induced oxidative stress and apoptosis by ameliorating relevant intracellular pathways associated with lipid accumulation and endothelial dysfunction. Our study results suggest that AdipoRon may be a promising drug for the restoration of diabetic nephropathy by reducing lipotoxicity through the activation of adiponectin receptors and downstream targets through stimulation of the intracellular Ca^2+^/AMPK-LKB1/PPARα pathway and also by increasing ceramidase activity [24,25].

## 6. Conclusions and Future Perspectives

**Figure 1:**
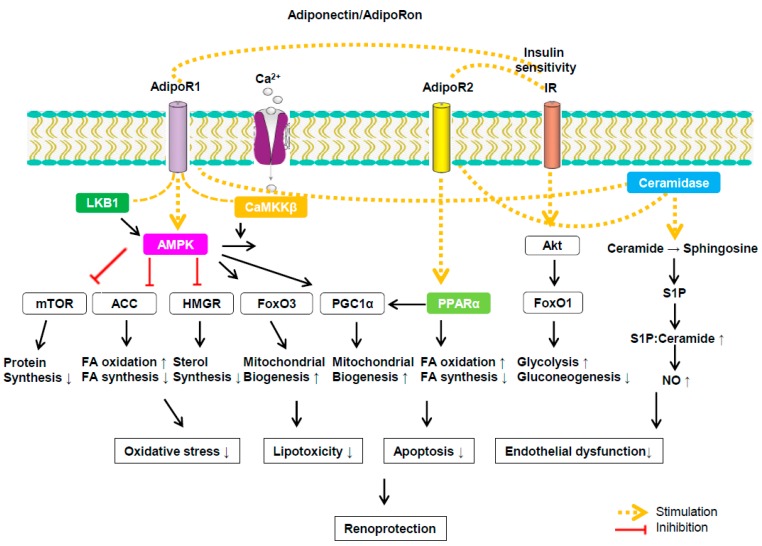
Signaling pathways associated with adiponectin and its receptor binding. AdipoR1 increases calcium influx to activate Ca^2+^/calmodulin-dependent protein kinase kinase β (CaMKKβ) and subsequent downstream kinases. AdipoR1 also activates liver kinase B1 (LKB1) and AMPK that can increase peroxisome proliferator-activated receptor (PPAR) gamma coactivator 1 alpha (PGC-1) expression. Activation of associated downstream pathways exert prometabolic effects by enhancing fatty acid oxidation and mitochondrial biogenesis. AdipoR2 activate PPARα to increase fatty acid oxidation and insulin sensitivity. AdipopR1/2 has ceramidase activity and can catalyze the conversion of ceramide to sphingosine, which produces sphingosine-1-phosphate (S1P), subsequently increasing S1P to ceramide ratio that further ameliorates endothelial dysfunction through increased NO level. Insulin-stimulated FoxO1 phosphorylation through PI3K and AKT can reduce hepatic gluconeogenesis.

The rationale for targeting adiponectin is based on the well-documented beneficial physiological actions of adiponectin spanning diabetes, inflammation, and metabolic diseases, and it is expected that studies in animal models will translate well to human physiology in the case of adiponectin. An excess intake of fatty acids can promote lipotoxicity and lipoapopotosis in several target organs, including diabetic kidneys. The binding of adiponectin to AdipoRs exerts renoprotective effects by regulating fatty acid metabolism; it enhances beta oxidation and ceramidase activity. AdipoRon can activate AdipoRs without affecting the systemic adiponectin level. It ameliorates lipotoxicity in DKD by increasing intrarenal AdipoRs that promote ceramidase activity and activates their downstream signaling, including the intracellular Ca^2+-^CaMKKβ/LKB1-AMPK/PPARα pathway (Figure 1). The discovery of the small molecular compound AdipoRon is an attractive therapeutic option that would mimic or enhance the established pro-metabolic actions of adiponectin but without the detrimental side effects due to chronic adiponectin exposure. Our recent studies highlight that targeting adiponectin receptors with low molecular weight agonists is a viable strategy, and that developing higher-affinity agonists with improved pharmacokinetics with a tissue- or cell-specific delivery approach should be pursued in the future.
